# Iridoids Isolation from a Phytochemical Study of the Medicinal Plant *Teucrium parviflorum* Collected in Iraqi Kurdistan

**DOI:** 10.3390/molecules27185963

**Published:** 2022-09-13

**Authors:** Fuad O. Abdullah, Faiq H. S. Hussain, Abdullah Sh. Sardar, Gianluca Gilardoni, Solveig Tosi, Giovanni Vidari

**Affiliations:** 1 Department of Chemistry, College of Science, Salahaddin University-Erbil, Erbil 44001, Kurdistan Region, Iraq; 2 Department of Pharmacognosy, Faculty of Pharmacy, Tishk International University, Erbil 44001, Kurdistan Region, Iraq; 3 Department of Medical Analysis, Faculty of Applied Science, Tishk International University, Erbil 44001, Kurdistan Region, Iraq; 4 Department of Biology, College of Education, Salahaddin University-Erbil, Erbil 44001, Kurdistan Region, Iraq; 5 Department of Chemistry, Universidad Técnica Particular de Loja, Loja 110107, Ecuador; 6 Department of Earth and Environmental Sciences, Mycology Laboratory, University of Pavia, 27100 Pavia, Italy; 7 Department of Chemistry, University of Pavia, 27100 Pavia, Italy

**Keywords:** *Teucrium*, Kurdish folk medicine, iridoid glycosides, harpagide, 8-*O*-acetylharpagide, biological activities

## Abstract

Herbal medicines are still widely practiced in Kurdistan Region-Iraq, especially by people living in villages on mountainous regions. Among plants belonging to the genus *Teucrium* (family Lamiaceae), which are commonly employed in the Kurdish traditional medicine, we have analyzed, for the first time, the methanol and aqueous methanol extracts of *T. parviflorum* aerial parts. The plant is mainly used by Kurds to treat jaundice, liver disorders and stomachache. We aimed to determine the phytochemical profile of the extracts and the structures of the main components, so to provide a scientific rationale for the ancient use of the plant in the ethno-pharmacological field. TLC analysis of the two extracts on silica gel and reversed phase TLC plates, using different visualization systems, indicated similar contents and the presence of phenolics, flavonoids, terpenoids and sugars. The chlorophyll-free extracts exhibited weak/no antimicrobial activities against a panel of bacteria (MICs = 800–1600 µg/mL) and fungal strains (MICs ≥ 5 mg/mL). At the concentration of 600 µg/mL, the methanol extract showed moderate antiproliferative effects against A549 and MCF-7 cancer cell lines in the MTS assay. Moreover, both extracts exhibited a significant dose-dependent free radical scavenging action against the 2,2-diphenyl-1-picrylhydrazyl (DPPH) radical (EC_50_ = 62.11 and 44.25 μg/mL, respectively). In a phytochemical study, a high phenolic content (77.08 and 81.47 mg GAE/g dry extract, respectively) was found in both extracts by the Folin–Ciocalteu assay. Medium pressure liquid chromatographic (MPLC) separation of the methanol extract on a reversed phase cartridge eluted with a gradient of MeOH in H_2_O, afforded two bioactive iridoid glucosides, harpagide (**1**) and 8-*O*-acetylharpagide (**2**). The structures of **1** and **2** were established by spectral data, chemical reactions, and comparison with the literature. Interestingly, significant amounts of hepatotoxic furano *neo*-clerodane diterpenoids, commonly occurring in *Teucrium* species, were not detected in the extract. The wide range of biological activities reported in the literature for compounds **1** and **2** and the significant antiradical effects of the extracts give scientific support to the traditional use in Iraqi Kurdistan of *T. parviflorum* aerial parts for the preparation of herbal remedies.

## 1. Introduction

**Figure 1:**
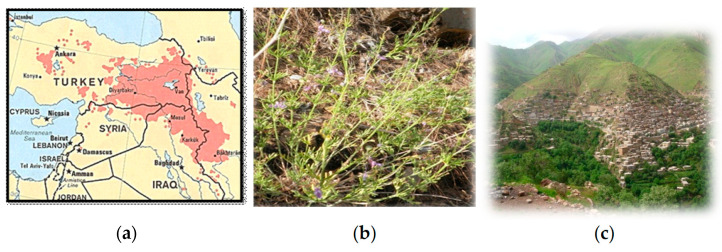
(**a**) an approximate map (pink area) of Kurdistan (taken and adapted from https://en.wikipedia.org/wiki/Iraqi_Kurdistan); (**b**) aerial parts of *T parviflorium*; (**c**) Khalafy mountain where the plant was collected (photos taken by one of the author, F.O.A.).

Nature is a major source of current medicines, and many (semi)synthetic drugs have been developed from the study of bioactive compounds isolated from extracts of plants used in traditional medicines of different countries [1]. The use of herbal remedies is practiced in Iraqi Kurdistan (Figure 1a) since time immemorial. Proof of this is the finding of pollens of eight different medicinal plants, still growing in Iraqi Kurdistan, in the soil samples of an approximately 60,000 years old Neanderthal burial at Shanidar IV cave in Northern Iraq [2]. The popularity of traditional medicines has increased among the Kurdish society in the last 20 years, not only in rural areas but also in main towns where different medicinal plants are sold at local bazaars [3]. Thus, the first choice for treating many diseases is the use of traditional plants [4,5]. This finding is due to the medicinal use practices being a part of the country’s cultural heritage and to the high cost of synthetic drugs, most of which are produced and imported from abroad.

We envisaged that a higher use of local medicinal plants would have obvious economic benefits to the people of Iraqi Kurdistan and may stimulate the development not only of small pharmaceutical and medicinal industries, but also the manufacture of other important products, for example, cosmetics, agrochemicals, nutraceutical ingredients, and food additives. Thus, these important aspects must seriously be considered in any industrial strategy aimed at improving the economies of developing countries, such as Iraqi Kurdistan [6,7]. Moreover, cultivated medicinal plants are renewable resources that facilitate the conservation of the biodiversity and ecological habitats.

Among medicinal plants occurring in the Kurdish traditional medicines *Teucrium* species turns out to be widely used. This finding is not unexpected because this is one of the most economically and ecologically important genera in the family Lamiaceae.

*Teucrium* plants are mainly distributed in mild climate regions; in fact, about 90% of the total *Teucrium* taxa grow in the Mediterranean and the Middle East Asian regions [8]. Moreover, they occur in America and Australia. Most *Teucrium* species have been used in ethnopharmacology for thousands of years, especially in countries ranging from North Africa to Middle East and South-Western Asia. The species have different potential applications, from pharmaceutical to food industries, primarily due to the high content of bioactive specialized metabolites. These include monoterpenes, sesquiterpenes, diterpenes, sterols, saponins, iridoids, flavonoids, polyphenolic compounds, fatty acids, alkaloids, and essential oils. Noteworthy, several compounds exhibited significant antipyretic, diuretic, diaphoretic, genotoxic, antioxidant, antibacterial, antifungal, antiviral, anticancer, cholesterol-lowering, hypoglycemic, anti-malaria, spasmolytic, anti-inflammatory, and even antifeedant effects [9,10,11,12,13].

Seven *Teucrium* taxa grow in the wild in Iraqi Kurdistan where they are used as herbal remedies, as discussed in a recent review [13].

*Teucrium parviflorum* Schreber, commonly called “chaqchaqa” in Kurdistan, grows wildly in the forests on high mountains, mainly in the districts of Amadiya, Sulaymaniya, and Rawanduz [14,15] of Iraqi Kurdistan, where the flowering months are May and June. The consume of this plant is especially quite common in the areas near the small town of Tawella, in the Sulaymaniyah Province. An aqueous infusion of aerial parts (Figure 1b) is prepared to treat jaundice, liver disorders, stomachache, and to reduce cholesterol level in blood. Outside Kurdistan, a decoction of aerial parts of *T. parviflorum* is utilized in Turkey in a popular treatment against hemorrhoids [16].

Despite the medicinal uses of *T. parviflorum*, little is known about the phytochemicals and biological activities of this plant. The few reported scientific investigations are related to the high antioxidant activities of ethanol and aqueous extracts of the plant [17]. Moreover, an essential oil steam-distilled in a Clevenger apparatus from aerial parts collected in Turkey was analyzed by GC and GC/MS techniques [18]. In this case, 33 components, representing 80.7% of the oil, were identified. The main components were the sesquiterpenoids β-caryophyllene (18.6%), germacrene D (9.2%), caryophyllene oxide (8.8%), and bicyclogermacrene (6%) [18].

Contrary to the essential oil constituents, the non-volatile specialized metabolites of *T. parviflorum* aerial parts were not yet isolated and identified at the onset of our work. Therefore, in continuation of our research project on the study of Iraqi Kurdistan medicinal plants, we examined the contents of polar extracts of *T. parviflorum* aerial parts. Our objectives were: (i) to estimate the antibacterial, antifungal, cytotoxic, and antiradical scavenging potential of the extracts through in vitro assays; (ii) to perform a preliminary phytochemical analysis of the extracts; (iii) to isolate and identify the main specialized metabolites occurring in the extracts; (iv) to investigate the possible presence of hepatotoxic *neo*-clerodane diterpenoids [19], considering the traditional use of *T. parviflorum* to cure liver and stomach diseases; (v) ultimately, to provide a phytochemical rationale for the ancient use of the plant in the ethno-pharmacological field. The results of our investigation are reported below.

## 2. Results and Discussion

Powdered air-dried aerial parts of *Teucrium parviflorum* collected in Kurdistan were thoroughly defatted by soaking in hexane at 25 °C; successively, considering the traditional method of use of the plant, the biomass was extracted with polar solvents, i.e., MeOH, followed by MeOH/H_2_O (70:30). Evaporation of the two extracts afforded residues B1 and C1 with yields of 6.0 and 5.58% (*w*/*w* dry aerial parts), respectively.

**Figure 2:**
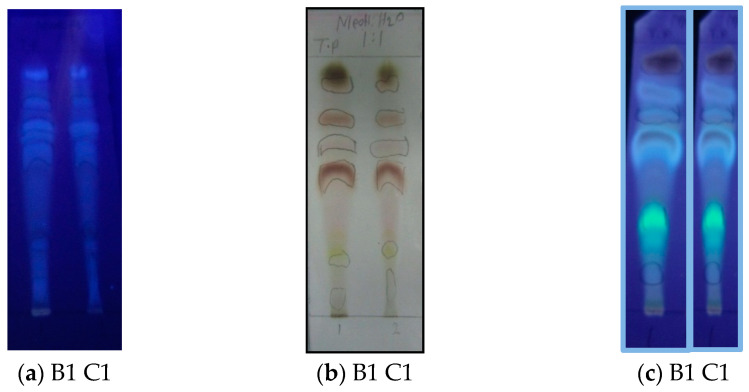
Analysis of B1 and C1 extracts on TLC plates (Merck RP-18 reversed phase; mobile phase: MeOH: H_2_O (1:1). Visualization systems: (**a**) UV light (366 nm); (**b**) vanillin/H_2_SO_4_ spray reagent; (**c**) NH_3_ fumes, followed by exposure to UV light (366 nm).

B1 and C1 were preliminary analyzed by TLC on silica-gel and reversed phase plates. Different visualization systems (see Section 3.4) were used, specific for different classes of natural products [20,21,22]. The chromatograms indicated similar contents in the two extracts and mainly the presence of terpenoids, phenolics, flavonoids, and sugars. Representative examples of TLC chromatograms are shown in Figure 2.

**Figure 3:**
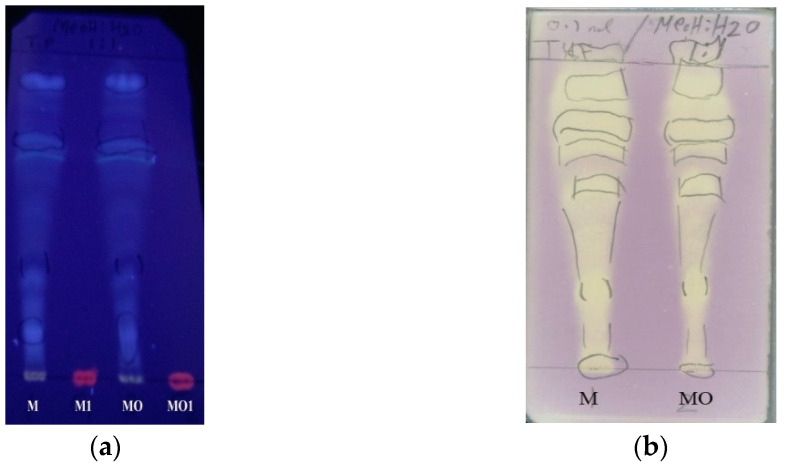
TLC analysis on a RP-18 reversed phase plate; mobile phase: MeOH: H_2_O (1:1). (**a**) The results of chlorophyll removal from residues B1 and C1; detection systems: UV light (366 nm). M and MO: chlorophyll-free methanol and aqueous methanol extracts, respectively; M1 and MO1: chlorophyll residues; (**b**) TLC analysis of M and MO residues by spraying a DPPH solution.

Subsequently, samples of B1 and C1 were separately filtered through an SPE C-18 cartridge to remove chlorophylls (revealed as a red fluorescent spot on TLC plates under UV light, see Figure 3a and other highly lipophilic compounds.

A preliminary qualitative test for the antiradical activity of the resulting chlorophyll-free fractions M and MO was then performed on TLC plates sprayed with a DPPH free radical solution. In fact, DPPH shows a strong absorption band at about 515 nm due to its odd electron and solution appears a deep violet color; the absorption vanishes as the electron pairs off by electron donation from an antioxidant. In the test, a long strip of whitish spots on a light-violet background appeared on the TLC plates, revealing that most components of the fractions M and MO had a remarkable radical scavenging activity (Figure 3b. Therefore, before conducting a quantitative estimation of the extract antiradical properties, the fractions M and MO were filtered through a Sephadex LH-20 column to remove possible tannin contaminants and polymeric material. Two residues, B1′ and C1′, were obtained in yields of 85 and 90%, respectively, by elution of M and MO, respectively, with aqueous MeOH, whereas elution with aqueous Me_2_CO, to recover high molecular weight compounds from the column, afforded residues B1″ and C1″, respectively, in yields of 7.8 and 7.2%, respectively. TLC analysis of the fractions (not shown) indicated the absence of condensed tannins in B1″ and C1″.

The following experiments were then conducted on partially purified residues B1′ and C1′.

**Figure 4:**
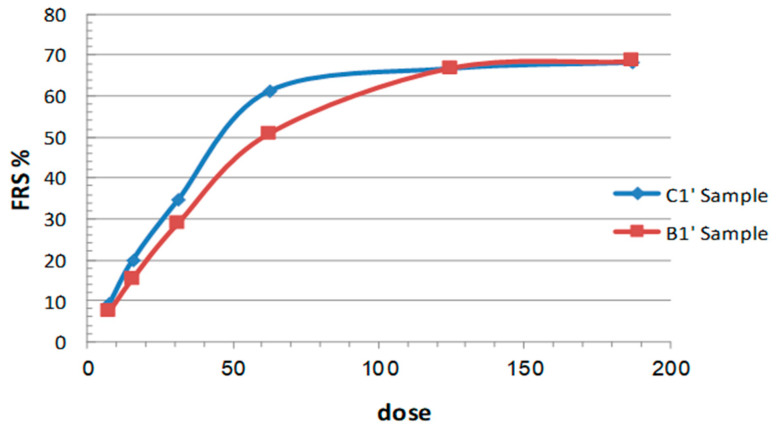
Percent free radical scavenging (FRS%) activity vs. sample concentration (μg/mL) of the extracts B1′ and C1′ in the DPPH test.

As envisioned by the presence of phenolic constituents, the extracts B1′ and C1′ exhibited significant dose-dependent free radical scavenging (FRS) effects (%) in the classical DPPH assay (Figure 4) [23]. The calculated EC_50_ values, i.e., the concentration of the sample required to reduce the initial DPPH concentration by 50% were equal to 62.11 ± 1.54 for B1′ and 44.25 ± 1.12 μg/mL for C1′, respectively. In comparison, the calculated EC_50_ of the positive control, α-tocopherol, was 9.09 ± 0.077 μg/mL. On the other hand, the total (poly)phenolic contents in B1′ and C1′ dry extracts, estimated in terms of gallic acid equivalent by means of the standard Folin–Ciocalteu assay procedure [24], were 77.08 (±0.06) and 81.47 (±1.32) mg GAE/g dry extract, respectively. These results indicated a direct correlation between the extract antiradical scavenging activity and the (poly)phenolic content of the extracts, which may be the foremost contributors to the antioxidant activity of the plant [17]. Phenolics are common constituents of *Teucrium* extracts. They mainly include glycosidic and aglycone forms of the flavones luteolin and apigenin, and phenylethanoid glycosides, such as verbascoside derivatives [13].

The antimicrobial and cytotoxic activities of the extracts were then measured by in vitro assays. A very weak activity was determined for B1′ and C1′ [MICs (Minimum Inhibitory Concentrations) = 800 and 1288 µg/mL, respectively] [25] against *Staphylococus pyogenes*, which is the major causative agent of skin purulent lesions, including erysipelas. Instead, the two extracts exhibited no significant antimicrobial effects [25,26,27] against the bacteria *Staphylococus aureus*, *Staphylococus mutans*, and *Escherichia coli* (MICs > 1600 µg/mL). On the other hand, the MBCs (Minimum Bactericidal Concentrations) of B1′ and C1′ were >1600 µg/mL against each of the four bacterial strains. For comparison, the corresponding values determined for gentamicin were in the range of 0.25–1 µg/mL. 

B1′ and C1′ were also inactive against important human fungal pathogens (*Candida albicans*, *Microsporum canis*, and *Trichophyton rubrum*), a plant pathogen (*Pyricularia grisea*), and a common biodeteriogenous species (*Aspergillus niger*) [26,27]. In fact, the MICs of B1′ and C1′ were ≥5 mg/mL against each of the five fungal strains, at least a thousand times higher than the reference antifungal agents (Table 1).

The antimicrobial inactivity of B1′ and C1′ was not unexpected considering that *T. parviflorum* is not used against infections in the traditional medicine of Kurdistan.

The antiproliferative activity of residue B1′ against MCF-7 (breast cancer) and A549 (lung cancer) cell lines was estimated by measuring cell viability using a microculture tetrazolium assay [28,29,30]. At the concentration of 600 µg/mL, the residue reduced the A549 and MCF-7 cell viability by about 55% and 27%, respectively. On the other hand, at the concentration of 60 µg/mL, the cell viability was only reduced by 14.8% and 14.5%, respectively. In comparison, the IC_50_ (µM) ± S.D values of cisplatin, used as the cytotoxic reference agent, were 17 (±4) and 13 (±2) against MCF-7 and A549 cells, respectively. Given the low antiproliferative activity of the extract B1′, that of isolated compounds (see below) was not tested. 

A sample (0.5 g) of residue B1′ was finally separated on a medium pressure liquid chromatography (MPLC) using a reversed-phase C-18 cartridge. Elution with a gradient of MeOH in H_2_O afforded 22 main fractions (B1′1-B1′22, in elution order) which were analyzed by TLC and NMR spectroscopy. The fractions B1′1 (174 mg), B1′2 (16 mg) and B1′3 (4.5 mg) contained mainly mixtures of sugars; the fractions B1′5 (5 mg), B1′6 (21 mg), and B1′7 (4 mg) were constituted by the iridoid harpagide (**1**) [31,32] with different degrees of purity; the fractions B1′8 (19.7 mg), B1′9 (29 mg), B1′10 (58 mg) and B1′11 (7 mg) were constituted by the iridoid 8-*O*-acetylharpagide (**2**) [31,33] (see NMR spectra in Supplementary Materials) with different degrees of purity; the fractions from B1′12 to B1′22, for a total of 94 mg, contained mainly mixtures of phenolic derivatives which were not analysed. 

**Figure 5:**
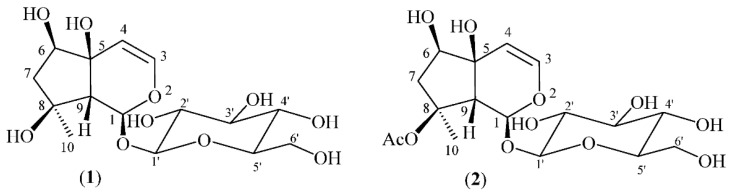
Chemical structures of harpagide (**1**) and 8-*O*-acetylharpagide (**2**).

The structures of harpagide (**1**, Figure 5), colorless powder, [α]_D_^22^−145 (MeOH), and 8-*O*-acetylharpagide (**2**, Figure 5), colorless powder, [α]_D_^22^−115 (MeOH), were determined by analysis of the MS, IR, and NMR spectra (Table 2) that corresponded in full to the literature data [31,32,33]. Moreover, separate acidic hydrolysis of **1** and **2** gave D-glucose, while basic hydrolysis of **2** afforded **1**. Isolated (unoptimized) yields of harpagide (**1**) were 5 and 4.25% (*w*/*w*), respectively, with respect to B1′ and the residue B1 from the methanolic extract of *T. parviflorum* aerial parts; the corresponding isolated (unoptimized) yields of 8-*O*-acetylharpagide (**2**) were 17.4 and 14.8% (*w*/*w*), respectively.

The iridoids **1** and **2** represent chemotaxonomic markers of the Lamiaceae family and have been identified in other *Teucrium* species distributed in Kurdistan [13] as well as in several genera of the Ajugoideae and Lamioideae subfamilies, for example *Ajuga*, *Stachys*, *Sideritis*, *Galeopsis*, *Melittis* [34]. Harpagide (**1**) is reported in the literature to exert a wide number of biological activities such as cytotoxic, anti-inflammatory, anti-osteoporotic and neuroprotective effects [35,36,37,38]. Similarly, in previous studies, 8-*O*-acetylharpagide (**2**) showed vasoconstrictor, antitumoral, antiviral, antibacterial and anti-inflammatory activities [39,40]. Moreover, the iridoid **2** exhibited an extraordinary ability to inhibit lipid peroxidation [41], and it was suggested that the presence of a free hydroxyl group on C5 of compound **2** could be responsible for the high antioxidant effects.

Contrary to the rather high contents of terpenoids **1** and **2**, *neo*-clerodane diterpenoids were not detected in significant amounts in the methanolic extract of *T. parviflorum* aerial parts. This finding is quite interesting because furano *neo*-clerodane diterpenoids probably represent the most abundant family of specialized metabolites occurring in *Teucrium* taxa and are considered the chemotaxonomic markers of the genus [42]. Moreover, several of them are probably the main responsible agents for acute and chronic hepatitis and even fatal cirrhosis affecting persons who consume teas or capsules prepared with the aerial parts of some *Teucrium* plants [13,19].

## 3. Materials and Methods

### 3.1. General Experimental Techniques and Procedures

Column chromatography (CC): Merck Kieselgel 60 (40–63 μm), and Merck LiChroprep RP-18 (25–40 μm); TLC: 0.25 mm Silica gel 60 (0.25 mm; GF254, Sigma-Aldrich, Steinheim, Germany) or RP-18 (F254, Merck), Al-supported plates; general spot visualization under UV light (254 and 366 nm). Semipreparative medium pressure liquid chromatographic (MPLC) separations were performed on an Isolera instrument (Biotage), equipped with a reversed phase cartridge (RP-C18 HS SNAP, 120 g) and a dual wavelength UV detector. Reagent-grade solvents, purchased from Carlo Erba (Milan, Italy) or from Aldrich, were used for extraction and chromatographic separations. Optical rotation: PerkinElmer 241 polarimeter. IR Spectra: PerkinElmer Paragon 100 PC FT-IR spectrometer; on KBr disks. NMR Spectra: Bruker AV300 spectrometer, at 300 (^1^H) and 75.47 MHz (^13^C). ^1^H NMR and ^13^C NMR chemical shifts are relative to signals of residual C*H*D_2_OD and *^13^C*D_3_OD in MeOH-*d*_4_ (Sigma-Aldrich, Steinheim, Germany) [*δ*_H_ 3.27 (central line of a quintuplet) and *δ*_C_ 49.0 (central line of a septuplet), respectively]; coupling constants (*J*) in Hz; the number of hydrogens bound to each C-atom was determined by DEPT experiments; COSY, DEPT, HSQC, NOESY spectra, were recorded using standard pulse sequences. ESI-MS: a Thermo-TSQ mass spectrometer, by flow injection analysis (FIA), with electron spray ionization source (ESI) at 5 kVon TIP capillary. HR-MS: FT-ICR Bruker-Daltonics Apex II mass spectrometer (Bremen, Germany).

### 3.2. Plant Material

Aerial parts of *Teucrium parviflorum* Schreb. (Figure 1b) were collected in the wild on the slopes of Khalafy Mountain (Tawela) (Figure 1c), in the south-east of Iraqi Kurdistan, near the border with Iran, on 28 June 2020, at an altitude of 1600 a.s.l. (GPS information: latitude 35°11′20.51″ N, longitude 46°11′34.58″ E). The plant was identified at the Education Salahaddin University Herbarium (ESUH) by the botanists Abdul Hussain Al Khayyat and Abdullah Saeed from the University of Salahaddin-Hawler/Iraq. A voucher specimen (No. TP 6855) has been deposited at the Salahaddin University herbarium (ESUH). After air-drying under shade at room temperature for a week, the raw material was finely powdered using a laboratory grinding mill and the resulting powder was stored in a glass bottle in a dark place until analysis.

### 3.3. Preparation of Defatted Extracts B1 and C1 from T. parviflorum Aerial Parts

Powdered aerial parts of *T. parviflorum* (550 g) were suspended in hexane (2.5 L), and the suspension was gently swirled at room temperature for 50 min in an ultrasonic bath. Subsequently, the mixture was decanted and filtered through a filter paper. This process was repeated 3 times; the liquids were brought together and the solvent was removed by a rotary evaporator to give a residue (A1, 2.88 g, 0.524% *w*/*w* dry vegetable material) which was stored in a vial at −5 °C, protected from light. Subsequently, the biomass was extracted with MeOH (2.5 L) in an ultrasonic bath for 1 h at room temperature for 1 h; subsequently, the mixture was decanted and filtered. This process was repeated 3 times, each time using fresh solvent (2 L). The liquids were then brought together and the volatiles were removed by evaporation under vacuum to afford a residue (B1, 33.0 g, 6.0% *w*/*w* dry vegetable material) that was stored in a bottle at −5 °C, protected from light, until use. Finally, the biomass was extracted for three times with a mixture of MeOH/H_2_O (70:30), 1.5 L each time, following the procedure previously described. The extracts were then brought together and after MeOH removal in a vacuum, the residual aqueous phase was lyophilized to give residue C1 (30.68 g, 5.58% *w*/*w* dry vegetable material) that was stored in a bottle at −5 °C until use.

### 3.4. Preliminary Phytochemical Analysis of Residues B1 and C1

The contents of defatted residues B1 and C1 were preliminary examined by analytical TLC under two chromatographic conditions: (a) RP-18 plates, eluted with MeOH/H_2_O (1:1); (b) silica-gel 60, eluted with EtOAc/*n*-BuOH/HCO_2_H/H_2_O (5:3:1:1). After drying, the plates were inspected using different visualization reagents [20,21,22]: (a) under UV light at 254 nm to reveal the presence of unsaturated and aromatic compounds; (b) under UV light at 366 nm before and after exposure to NH_3_ fumes to reveal the presence of phenolic compounds such as flavonoids ; (c) under visible light after spraying with a 5% solution of FeCl_3_ in 0.5N HCl to detect phenols; (d) under visible and UV light after spraying with an 1% solution of AlCl_3_ in EtOH to detect flavonoids; (e) with 0.5% vanillin or anisaldehyde solution in sulfuric acid/EtOH (4:1), followed by heating at 110 °C for about 1 min, for the detection of sugars, terpenes, steroids, etc. [22,43]; (e) with a 0.1 mM solution of 2,2-diphenyl-1-picrylhydrazyl (DPPH) in EtOH to detect radical scavenging antioxidants. 

### 3.5. Chromatographic Separations of Residues B1 and C1

A sample of residue B1 (1.0 g) was filtered through a solid-phase extraction C-18 cartridge (Discovery^®^ DSC-18 SPE Tube, bed wt. 10 g, volume 60 mL, MilliporeSigma™ Supelco™ 52609U, Sigma-Aldrich, Steinheim, Germany) previously conditioned with MeOH (100 mL), followed by H_2_O (100 mL). The sample was dissolved by stirring in MeOH/H_2_O (90:10) in an ultrasonic bath for 1 h, loaded on the top of the column, and eluted by 200 mL of MeOH/H_2_O (90:10). The filtrate was evaporated to dryness under vacuum. Subsequently, the resulting residue M (0.96 g) was filtered with a flow of 4 mL/min through a Sephadex LH-20 column (45 g, Sigma) that had previously been swollen in MeOH/H_2_O (80:20) overnight. The sample was dissolved in 10 mL of MeOH/H_2_O (80:20) in an ultrasonic bath for 1 h and it was then loaded on the column. Elution with equal volumes (300 mL each) of MeOH/H_2_O (80:20), MeOH/H_2_O (65:35), MeOH/H_2_O (50:50), in the order, followed by evaporation of eluates to dryness under vacuum gave residue B1′ (850 mg). Subsequently, fraction B1″ (78 mg), containing high MW compounds was eluted from the column with Me_2_CO/H_2_O (80:20). An analogous experiment conducted on a sample (0.5 g) of extract C1 afforded residues C1′ (450 mg) and C1″ (36 mg).

A sample of residue B1′ (0.5 g) was separated on MPLC instrument using a commercial reversed-phase C-18 cartridge. The mobile phase was a mixture of H_2_O (A) and MeOH (B). A linear gradient was applied from an A/B mixture (80:20) to 100% B, over 2 h at room temperature, at a flow rate of 10 mL/min; dual UV detection wavelengths were set at 210 and 254 nm, and fractions of 10 mL each were collected; subsequently, the solvent in each tube was evaporated using a liquid nitrogen trap and a centrifuge under vacuum, and the resulting residue was weighed. The overall recovery of the chromatographed sample was 89.3%. Subsequently, the content in each fraction was analyzed by TLC on analytical silica gel 60 plates, eluted with *n*-hexane/EtOAc (80:20), and on RP-18 plates, eluted with MeOH/H_2_O, (1:1) and MeOH/H_2_O (3:1). Spots were detected under UV light at 254 and 366 nm and by spraying the plates with 0.5% vanillin in EtOH/sulfuric acid (1:4) [43], followed by heating at 100 °C for about 0.5 min. Fractions with homogeneous content were grouped together to give 22 main fractions, numbered from B1′1 to B1′22, which were analyzed by NMR spectroscopy. Harpagide (**1**) (25 mg) was isolated in a pure form from fractions B1′6 and B1′7 and 8-*O*-acetylharpagide (**2**) (87 mg) was isolated in a pure form from fractions B1′9 and B1′10.

### 3.6. Alkaline Hydrolysis of Iridoid ***2*** to Give ***1***

Iridoid **2** (3 mg) was dissolved in a saturated solution of K_2_CO_3_ in MeOH (1.5 mL) and the solution was stirred for 10 h at room temperature. H_2_O (10 mL) was added; the layer was carefully neutralized with 10% HCL and extracted with EtOAc (3 × 7 mL). The organic phase was dried (Na_2_SO_4_) and evaporated to give a residue (2 mg) that was identical to harpagide (**1**).

### 3.7. Acid Hydrolysis of Iridoids ***1*** and ***2***

Iridoids **1** and **2** (2.0 mg each) were separately dissolved in 2N CF_3_COOH (1.5 mL) in a sealed vial and heated at 100 °C for 6 h. After cooling to room temperature, dilution with H_2_O (5 mL), neutralization with NaOH, and exhaustive extraction with EtOAc, the aqueous layers were repeatedly evaporated under vacuum to dryness with the aid of added MeCN. The resulting residues, which showed a positive optical rotation, were identified with an authentic sample of D-glucose by silica gel TLC, using CHCl_3_/MeOH/H_2_O (16:9:2) as the eluent [44].

### 3.8. Anti-Radical Activity Test

The Free Radical Scavenging Activities (FRSAs) of residues B1′ and C1′ were determined by the 2,2-diphenyl-1-picrylhydrazyl stable radical (DPPH, Sigma-Aldrich, Steinheim, Germany) method [23], using α-tocopherol (Sigma-Aldrich, Steinheim, Germany) as the reference standard. For each sample, separately dissolved in MeOH, six stock solutions were prepared having a concentration of 7.5, 5.0, 2.5, 1.25, 0.6, and 0.3 mg/mL, respectively. Subsequently, for each sample, test solutions at concentrations of 187.5, 125, 62.5, 31.25, 15, and 7.5 μg/mL, respectively, were prepared by adding 100 µL of each stock solution to 3.9 mL of a DPPH solution. The free radical solution was freshly prepared dissolving DPPH in methanol/KH_2_PO_4_ and NaOH buffer (50/50 *v*/*v*) at a concentration of 6 × 10^−5^ M (pH 7.4). After 20 min of incubation at room temperature in the dark, the absorbance of each test solution was measured at 515 nm using a UV-Visible spectrophotometer (Lambda 25 UV/VIS spectrometer N. 3903, Perkin Elmer instruments, Boston, MA, USA). The values of the Percent Free Radical Scavenging (FRS%) activity were expressed as the percent reduction of the DPPH radical by the B1′, C1′, and α-tocopherol test solutions, respectively, compared with the control, consisting of the DPPH solution (3.9 mL) and MeOH (100 µL). Different equations are reported in the literature to calculate FRS%. In our case, the following formula was used: (FRS%) = [(A_control_ − A_s__ample_)/A_control_] × 100. The analyses were carried out in triplicate and the results were expressed as the means ± SD. 

### 3.9. Determination of the Total Phenolic Content in B1′ and C1′ by the Folin–Ciocalteu Assay

The total phenolic content in the extracts B1′ and C1′, respectively, was determined by the Folin–Ciocalteu assay [24]. 10% EtOH was the solvent for the standard and the samples. A calibration line, absorbance vs. concentration, was constructed using gallic acid (Sigma-Aldrich, Steinheim, Germany) as the standard at various concentrations where linearity was observed. The equation of the calibration curve was y = 0.0876 (7) x + 0.006 (2), R^2^ = 0.996. Extracts B1′ and C1′ were diluted at various concentrations so that the corresponding absorbances fell within the range of the calibration curve of the standard. Absorbances were read at 760 nm, against a blank containing 1 mL of 10% EtOH. Analyses were conducted in triplicate, and the means and the standard deviations (SD) were calculated. The total phenolic content in each sample was determined using the formula: C = c V/m, where C = total phenolic content, expressed as mg gallic acid equivalents (GAE)/g dry extract, c = concentration (mg/mL) of gallic acid obtained from the calibration curve, V = volume (mL) of extract, m = mass (g) of extract.

### 3.10. Antibacterial Activity Assay

The antibacterial activities of residues B1′ and C1′ against *Staphylococus aureus* (ATCC 6538), *Staphylococus mutans* (ATCC 25175), *Staphylococus pyogenes* (ATCC 19615), and *Escherichia coli* (ATCC 10356) were expressed as the Minimum Inhibitory Concentration (MIC) and the Minimum Bactericidal Concentration (MBC) against each strain. The MIC is defined as the lowest concentration (µg/mL) of an antimicrobial that inhibits the visible growth (no turbidity is seen) of a microorganism after overnight incubation in a test tube, whereas the lowest concentration (µg/mL) of an antimicrobial with no visible growth of bacterial cells is defined as the MBC, indicating 99.5% killing of the original inoculum. It is determined by subculturing the last clear MIC tube onto the antibiotic-free growth medium and examining the bacterial growth. Gentamicin was used as the positive control. Each strain was first incubated overnight (18–20 h) in the cultural medium Tryptone Soya Broth (TSB; Oxoid LTD., Basingstoke, Hampshire, England) at 37 °C. The culture was then centrifuged at 3000 rpm for 20 min to separate the cells from the culture medium. Separated cells were resuspended in Iso-Sensitest Broth (ISB; Oxoid LTD, Basingstoke, Hampshire, UK), and the bacterial suspension was appropriately diluted to obtain an absorbance of 0.2 at λ = 655 nm, measured by a spectrophotometer (Jasco-320 Uvidec Japan Spectroscopic Co., Ltd., Tokyo, Japan), which corresponded to a concentration of microorganisms in the range of 10^7^–10^8^ CFU/mL.

The MIC of each extract was determined by the method of dilution with culture medium [25]. ISB series of tubes containing constantly increasing concentrations of B1′ or C1′ were inoculated with the same amount of each bacterial suspension. After incubation for 18–24 h at 37 °C, the MIC was determined by turbidity and expressed as μg of the extract used in the assay/total volume [volume of microbial rich medium (mL) + volume antimicrobial agent (mL)] in the tube. The MBC values were calculated by inoculating aliquots of culture medium taken from the tubes where the MICs were determined, in test tubes containing extract-free culture medium. Three replicates were performed for each test. 

### 3.11. Antifungal Activity Assay

The antifungal activity of extracts B1′ and C1′ were determined by means of the microdilution method [26,27] in 96-well microtiter plates (Cellstar, Greiner bio-one) against *Candida albicans* Berkhout (LM 460) and *Aspergillus niger* Tiegh. (LM 455) strains. B1′ was also tested against *Microsporum canis* E. Bodin ex Gueg. (LM 471), *Pyricularia grisea* Sacc. (LM 483), and *Trichophyton rubrum* (Castell.) Sabour (LM 502) strains. The fungi are deposited in the collection of the Mycology Laboratory at the University of Pavia with the indicated LM numbers. Fluconazole was used as the reference antifungal agent. The lowest concentration without visible fungal growth (at the binocular microscope) was defined as the concentration that completely inhibited fungal growth (MIC = Minimum Inhibitory Concentration).

The fungi were cultured until suitable conidiation and maintained at 28 °C on Sabouraud (SAB) agar medium (1 L of distilled water, 30 g of glucose, 10 g peptone). Fungal suspensions, at a concentration of about 10^5^ CFU/mL, were prepared from a 24-h-old culture in the case of *C. albicans* and one-week-old cultures in the case of the other strains. After having verified the extracts’ solubility, serial dilutions of each extract were prepared using the SAB medium inoculated with the same amount of the tested fungus. The final sample concentrations were 20, 10, 5, 2.5, 1.25, 0.6, 0.3, 0.15, 0.07 mg/mL. A volume of 50 µL of each dilution was distributed in each well. Three replicates were performed for each sample and dilution. Inoculated microplates were incubated at 37 °C in the case of *C. albicans* and at 27 °C in the case of the other strains. The MIC was determined after 24 h for *C. albicans* and *Aspergillus niger*, and after 5 days for the other fungi.

### 3.12. Antiproliferative/Cell Viability Assay (MTS Test) for Residue B1′

The antiproliferativity/cell viability assay is based on the reduction of the yellow [3-(4,5-dimethylthiazol-2-yl)-5-(3-carboxymethoxyphenyl)-2-(4-sulfophenyl)-2H-tetrazolium] salt (MTS) by NAD(P)H-dependent dehydrogenase enzymes in metabolically active cells to a purple formazan salt, which is soluble in cell culture media [29,30]. The formazan salt has a maximum of absorbance at about 490 nm in the UV spectrum. The measure of the absorbance can be directly related to the number of viable (living) cells. MCF-7 breast cancer cells (ATCC No. HTB-22) and A549 lung cancer cells (ATCC No. CLL-185) were grown in flasks for several days in a CO_2_ incubator (Forma Scientific) at 37 °C under a humidified air added of 5% CO_2_, changing the liquid growth medium (RPMI 1640, Euroclone, Europe) supplemented with 10% fetal bovine serum (FBS, Gibco, Paisley, UK), and 0.005% l-glutamine, penicillin and streptomycin (Life Technologies, Milan, Italy) whenever needed. When a cell culture reached confluence, a small amount of trypsin was added to the medium to separate the cells from the flask; after 3 min of incubation at 37 °C, FBS (1 mL) was added to stop the action of trypsin and avoid cells’ membrane degradation. The cell-containing medium was then transferred into a cell strainer and centrifuged (ALC 4232 Centrifuge) at 1000 rpm for 10 min. The resulting pellet was resuspended in the growth medium (1 mL), and the cells were separated using an automatic pipette and counted using a counting chamber and trypan blue as a dye. Separated cells were plated in 96-well flat-bottom microtiter plates (Cellstar, Greiner bio-one) at a density of 5 × 10^3^ cells in 100 µL of growth medium in each well. After 2 h of incubation, the growth medium was replaced with 100 µL of test medium (RPMI 1640 added with 0.005% l-glutamine, penicillin and streptomycin) and the plate was incubated for 24 h. Test solutions (1 mL each) with concentrations equal to 600, 60, 6, 0.6, 0.06 µg/mL, respectively, were prepared by diluting stock solutions (60 mg/mL MeOH:DMSO, 1:2) of residue B1′ and cisplatin (as the positive control) with the test medium. The test medium in the wells was replaced with a solution (100 µL) of increasing sample concentration. The final MeOH/DMSO concentration in each well exhibited no significant interference with the biological activities tested. The microplates were again incubated for 24 h; subsequently, the sample containing medium was replaced with fresh test medium (100 µL) and 20 µL of MTS tetrazolium reagent (CellTiter 96^®^—AQueous One Solution Cell Proliferation Assay, Promega, Madison, WI, USA). After 2 h of incubation, the absorbance was measured at 490 nm using a plate reader (BioRAD Model 550 Microplate Reader, BioRad, Hercules, CA, USA). To establish a negative control, the absorbance at 490 nm was measured for wells containing cells treated with the same medium (100 µL) as that used to deliver test compounds, to which MTS (20 µL) was added. Hence, the negative control was set to 100% cell survival or 0% toxicity. The absorbance of the blank (‘no cells’) wells was subtracted from all other absorbance values. For each experiment, five replicates were performed.

To calculate the ratio of the number of dead cells to the number of viable (living) cells, the average absorbance of the wells containing the cells treated with the antiproliferative agent was divided by the average absorbance of the negative control.

## 4. Conclusions

Aerial parts of *T. parviflorum* are used in the traditional medicines of Kurdistan and Turkey. In this work, we have investigated, for the first time, the non-volatile constituents of defatted polar extracts of the aerial parts.

Phenolics and the iridoids harpagide (**1**) and 8-*O*-acetylharpagide (**2**) were found in significant amounts in the extracts. Moreover, a high radical scavenging activity was determined for the methanolic extract. Thus, a direct correlation was envisaged between the antiradical scavenging activity and the (poly)phenolic content of the extracts, which may be the foremost contributors to the antioxidant activity of the plant. Thus, *T. parviflorum* is a potential source of natural antioxidants that may provide precious functional ingredients useful for the prevention of diseases related to oxidative stress. Instead, the methanol extract of the aerial parts showed only moderate antiproliferative activity against two tumor cell lines and exhibited no antimicrobial effects *in vitro*. This finding was not unexpected considering that the plant is not used against infections and tumors in the traditional medicine of Kurdistan.

In conclusion, the presence of phenolics and iridoids **1** and **2**, for which a wide range of biological activities has been determined in previous studies [35,36,37,38,39,40,41], provides, at least in part, a phytochemical rationale for the ancient use of the plant in the ethno-pharmacological field.

We project to complete in the future the study of the main specialized metabolites from *T. parviflorum* aerial parts, especially flavonoids, and to develop a procedure for the quantitative isolation of the important bioactive iridoids harpagide (**1**) and 8-*O*-acetylharpagide (**2**). Moreover, our aim is to definitively exclude the presence of hepatotoxic *neo*-clerodane diterpenoids [19] in polar extracts, considering the traditional use of *T. parviflorum* in Kurdistan to cure liver and stomach diseases.

## Figures and Tables

**Table 1:** Antifungal activity (MICs) of *Teucrium parviflorum* Schreb. aerial part extracts compared to some well-known antifungal agents.

Sample	*Pyricularia grisea*	*Microsporum canis*	*Trichophyton rubrum*	*Aspergillus niger*	*Candida albicans*
MeOH extract	>5 mg/mL	>5 mg/mL	>5 mg/mL	5 mg/mL	>5 mg/mL
Aqueous MeOH extract	>5 mg/mL	>5 mg/mL			
Itraconazole				0.0005 mg/mL	0.004 mg/mL
5-Fluorocitosine				0.004 mg/mL	
Amphotericin B	0.001 mg/mL			0.0005 mg/mL	0.5 mg/mL
Voriconazole				0.0005 mg/mL	0.004 mg/mL

**Table 2:** ^1^H-NMR (300 MHz, MeOH-*d*_4_, TMS; *δ*_H_ in ppm, *J* in Hz) and ^13^C-NMR (75 MHz, MeOH-*d*_4_, TMS, *δ*_C_ in ppm) spectral data of harpagide (**1**) and 8-acetylharpagide (**2**) isolated from aerial parts of *Teucrium parviflorum*.

Proton/Carbon	Harpagide (1)		8-Acetylharpagide (2)	
	^1^H ^a^	^13^C ^b^	^1^H ^a^	^13^C ^b^
1	1H, 5.75 d (1.2)	93.5 (CH)	1H, 6.09 d (1.2)	94.8 (CH)
3	1H, 6.33 d (6.4)	142.8 (CH)	1H, 6.4 d (6.4)	144.1 (CH)
4	1H, 4.96 br d (6.4)	108.7 (CH)	1H, 4.93 br d (6.4, 1.1)	107.2 (CH)
5	−	72.7 (C)	−	73.6 (C)
6	1H, 3.69 br d (4.3)	77.8 (CH)	1H, 3.73 br d (4.5)	78.5 (CH)
7	7a 1H, 1.91 dd (13.6, 4.7);	47.5 (CH_2_)	7a 1H, 1.95 dd (15.0, 4.5);	46.4 (CH_2_)
	7b 1H, 1.81 dd (13.6, 1.0)		7b 1H, 2.19 br d (15.1)	
8	−	78.6 (C)	−	88.9 (C)
9	1H, 2.55 br s	59.9 (CH)	1H, 2.87 br s	55.8 (CH)
10	3H, 1.28 s	25.2 (CH_3_)	3H, 1.46 s	22.8 (CH_3_)
1′	1H, 4.59 d (7.9)	99.7 (CH)	1H, 4.61 d (7.9)	100.2 (CH)
2′	1H, 3.21 dd (8.0, 9.0)	74.6 (CH)	1H, 3.90 dd (12.0, 1.5)	74.8 (CH)
3′	1H, 3.34 t (9.0)	78.4 (CH) ^c^	1H, 3.30–3.37 m	77.9 (CH)
4′	1H, 3.30 t (9.0)	72.0 (CH)	1H, 3.22–3.28 m	72.0 (CH)
5′	1H, 3.28–3.35 m	78.5 (CH) ^c^	1H, 3.30–3.35 m	77.9 (CH)
6′	6′a 1H, 3.66 dd (12.2, 5.6);	63.1 (CH_2_)	1H, 3.68 dd (12.0, 5.5);	63.1 (CH_2_)
	6′b 1H, 3.91 dd (12.2, 1.5)		1H, 3.93 dd (12.0, 1.5)	
-OCO*Me*	−	−	3H, 2.02 s	22.5 (CH_3_)
-O*C*OMe	−	−	−	173.6 (CO)

^a^ Proton assignments are based on COSY and HSQC experiments. ^b^ The number of hydrogens attached to each carbon was determined by DEPT experiments. Carbon assignments were established by HSQC and HMBC experiments. ^c^ Assignments can be interchanged.

## Data Availability

See Supplementary Materials.

## Supplementary Materials

The following supporting information can be downloaded at: https://www.mdpi.com/article/10.3390/molecules27185963/s1, Figures S1 and S2: ^1^H- and ^13^C-NMR spectra of harpagide (**1**) and Figures S3 and S4: ^1^H- and ^13^C-NMR spectra 8-*O*-acetyl harpagide (**2**).
