# Supplementary material for: Iridoids Isolation from a Phytochemical Study of the Medicinal Plant Teucrium parviflorum Collected in Iraqi Kurdistan
Source: Molecules. 2022 Sep 13;27(18):5963. doi: 10.3390/molecules27185963 (PMC9502474; doi:10.3390/molecules27185963)
Supplement: Supplementary file 1 [file molecules-27-05963-s001.zip › molecules-1877033-supplementary.pdf]

Iridoids isolation from a phytochemical study of the medicinal plant *Teucrium parviflorum* collected in Iraqi Kurdistan

SUPPLEMENTARY MATERIALS

S1. ^{1}H-NMR spectrum of harpagide (**1**)
S2. ^{13}C-NMR spectrum of harpagide (**1**)
S3. ^{1}H-NMR spectrum of 8-*O*-acetylharpagide (**2**)
S4. ^{13}C-NMR spectrum of 8-*O*-acetylharpagide (**2**)

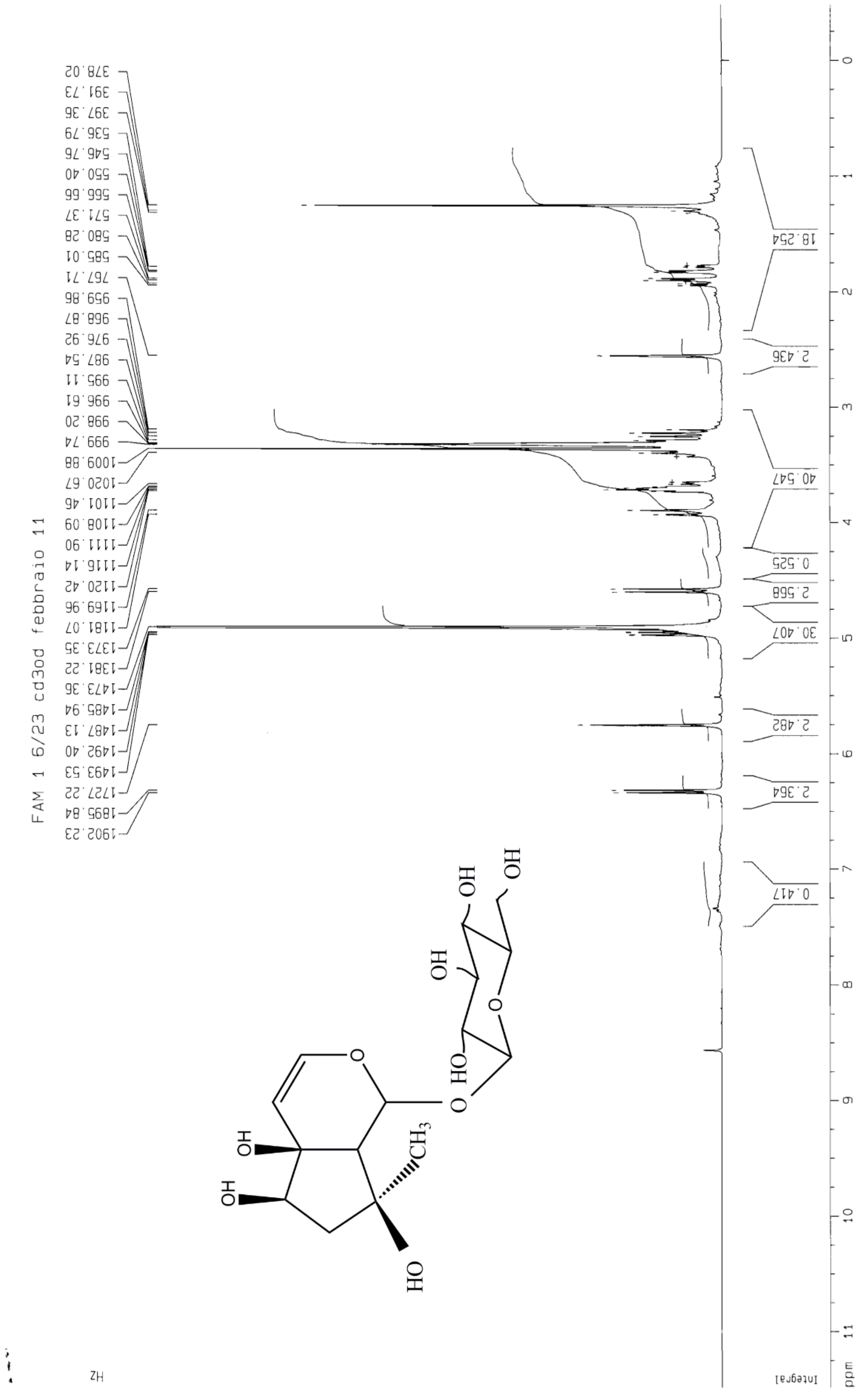

Figure S1. ^{1}H-NMR spectrum of harpagide (**1**) (MeOH-d_4, 300 MHz).

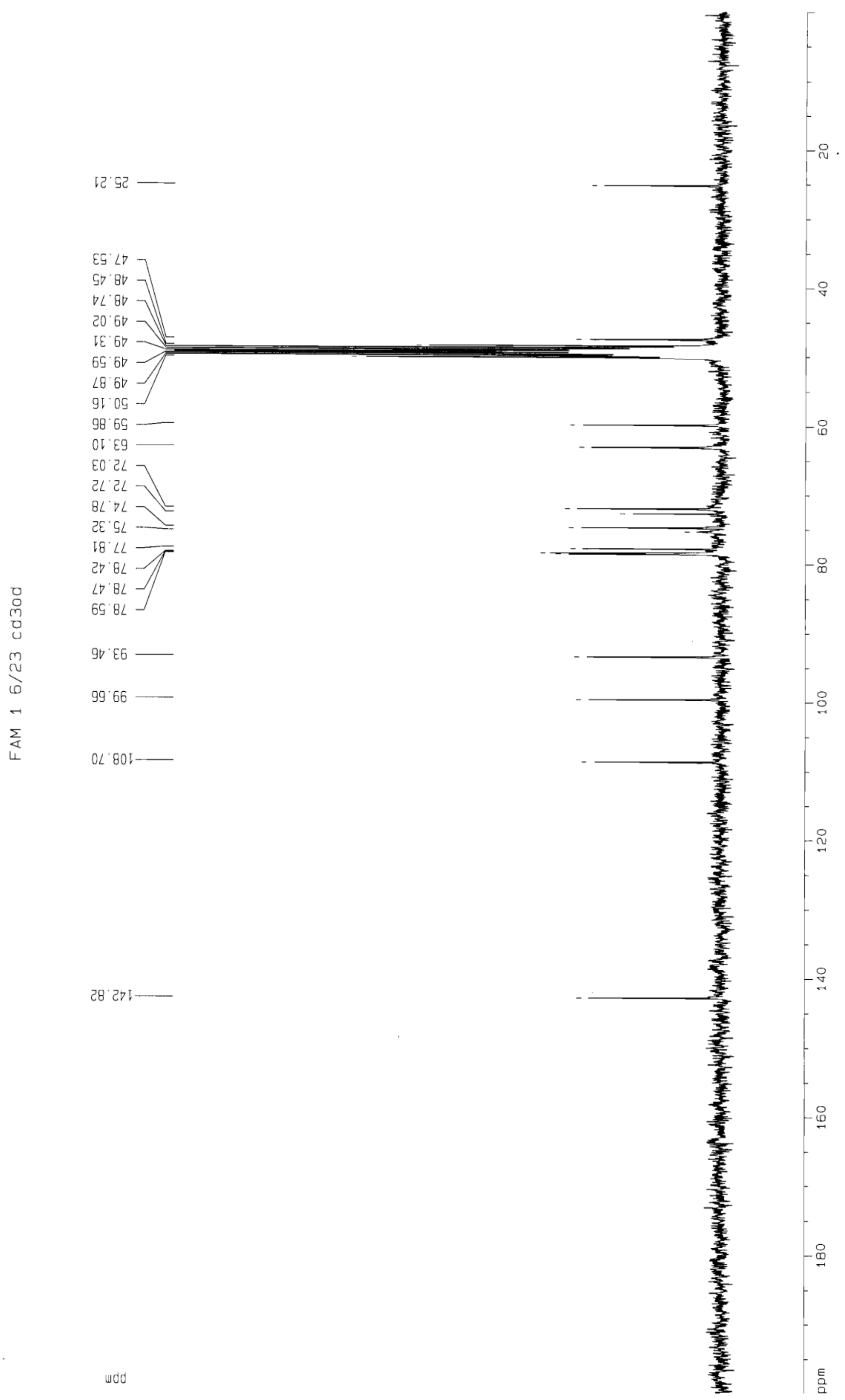

Figure S2. ^{13}C-NMR spectrum of harpagide (**1**) (MeOH-d_4, 75 MHz).

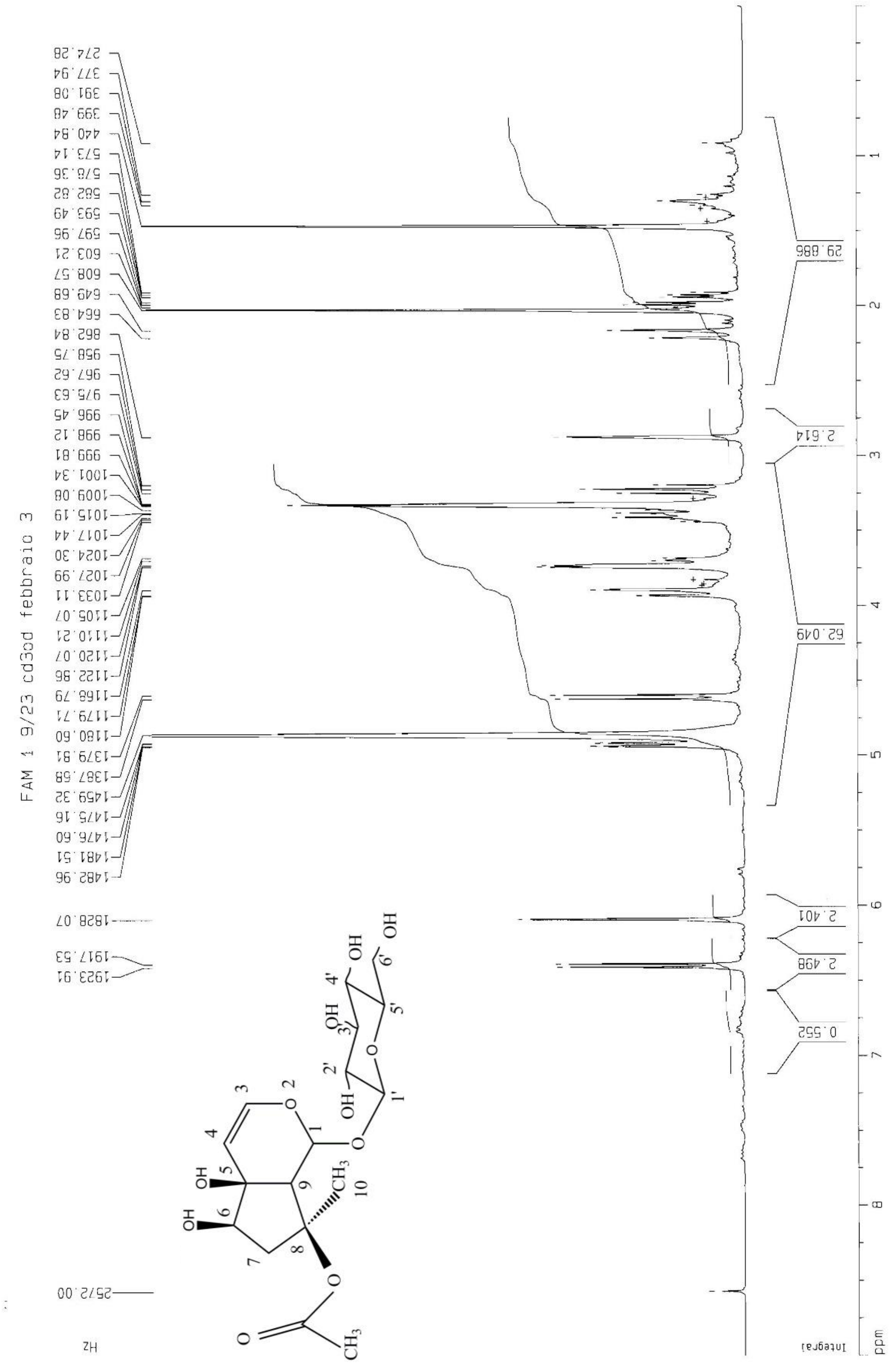

Figure S3. ^{1}H-NMR spectrum of 8-*O*-acetylharpagide (**2**) (MeOH-d$_4$, 300 MHz).

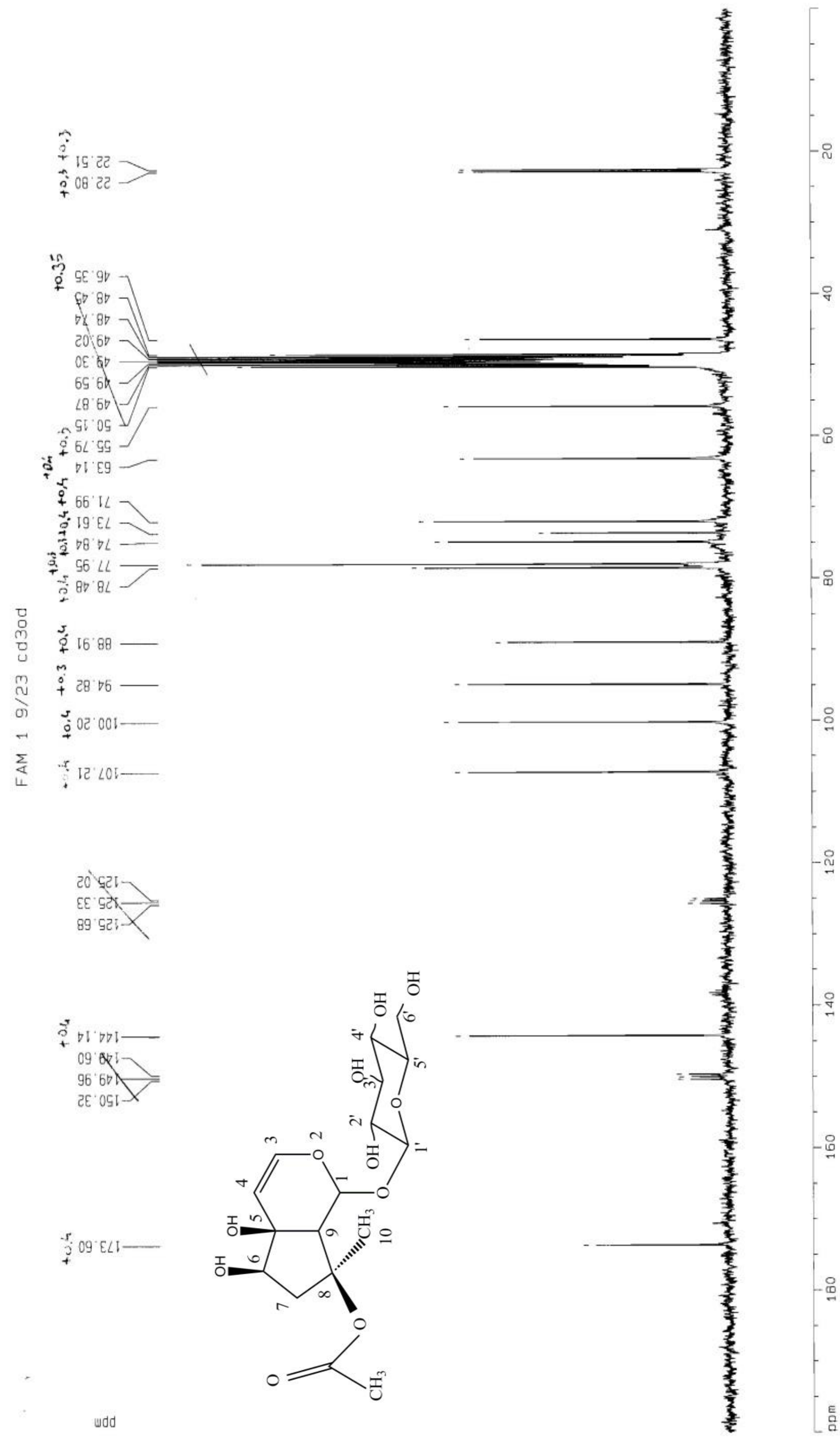

Figure S4. ^{13}C-NMR spectrum of 8-*O*-acetylharpagide (**2**) (MeOH-d$_4$, 75 MHz).
